# Starch Films Reinforced with Amazonian Manganese Ore Residues: Mechanical, Water Vapor Barrier, and UV-Shielding Performance

**DOI:** 10.1021/acsomega.5c05495

**Published:** 2025-11-26

**Authors:** João Otávio Donizette Malafatti, Simone Quaranta, Bruno Apolo Miranda Figueira, Gabriela Leite da Silva, Andressa Cristina de Almeida Nascimento, Alessio Mezzi, Alessandro Latini, Elaine Cristina Paris

**Affiliations:** † National Nanotechnology Laboratory for Agriculture (LNNA), Embrapa Instrumentação, São Carlos, São Paulo 13560-970, Brazil; ‡ 228703 Institute for the study of nanostructured material (ISMN), CNR, Montelibretti, Rome 00010, Italy; § Program of Science and Engineering of Materials, Federal University of Pará, Ananindeua, Pará 67130-660, Brazil; ∥ Department of Biology, 67828 Federal University of São Carlos, São Carlos, São Paulo 13565-905, Brazil; ⊥ Department of Chemistry, Federal University of São Carlos, São Carlos, São Paulo 13565-905, Brazil; # Department of Chemistry, Sapienza University of Rome, Rome, Rome 00185, Italy; △ Institute for the Study of Nanostructured Materials, Italian National Research Council (ISMN–CNR), Strada Provinciale 35d 9, 00010 Rome, Italy

## Abstract

Mining waste-derived reinforcing materials represent a sustainable strategy for enhancing the performance of packaging composites. Incorporating mining tailings and low-end nanomaterials synthesized from these residues into polymer-based films combines low-cost manufacturing with circular economy principles. In this study, manganese ore beneficiation waste and two manganese-based compounds synthesized from the same tailings, namely, manganese ore tailings (RBK), BaMnO_4_, and MnO_2_, were evaluated as reinforcing agents in casting starch films suitable for packaging applications (e.g., shopping bags). These materials were incorporated at concentrations ranging from 0.25 to 1% (w w^–1^). The addition of RBK and BaMnO_4_ significantly reduced the water vapor permeability (WVP) of the starch films from 4.9 ± 0.9 × 10^–10^ to 2.5 ± 0.5 × 10^–10^ and 2.8 ± 0.5 × 10^–10^ kg m^–1^ s^–1^ Pa^–1^, respectively. BaMnO_4_ also notably enhanced tensile strength, increasing it from 3.5 ± 0.2 to 20 ± 4 MPa, regardless of concentration. Additionally, 1% (w w^–1^) δ-MnO_2_ produced broad near-UV/visible attenuation, whereas RBK and BaMnO_4_ primarily strengthened the UVB barrier, these changes being accomplished with a higher opacity (600 nm). Overall, the incorporation of manganese-based materials derived directly from manganese ore beneficiation residues shows strong potential for improving the functional properties of starch films, enabling the development of low-cost, value-added, and environmentally responsible packaging materials. Furthermore, the reuse of mining waste contributes to mitigating the environmental impact associated with tailings storage.

## Graphical abstract

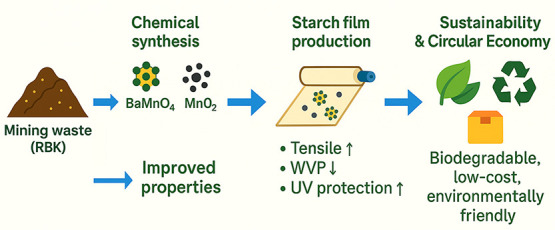

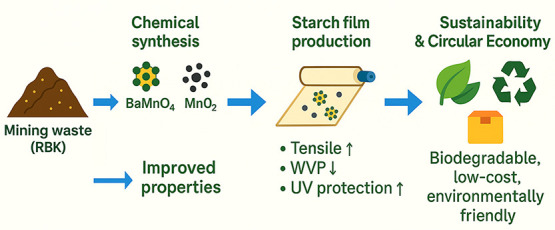

## 1 Introduction

Biopolymers have gained considerable attention as sustainable building blocks for developing low-cost, renewable, and environmentally friendly materials.^1^ Among the various biopolymers, starch stands out due to its abundance, biodegradability, and availability from agricultural sources such as corn, potatoes, and cassava.^2^ Consequently, recent research has focused on exploiting starch derived from agro-industrial residues, such as cassava peels, corn husks, and potato processing byproducts, to promote circular economy practices. Reusing these byproducts contributes to biomass valorization and minimizes environmental impact, while creating new value chains within the agricultural and food sectors.

Starch-based films have been proposed as biodegradable matrices for packaging, disposable bags, and even functional materials, such as controlled-release fertilizers. Despite these advantages, starch suffers from high hydrophilicity and limited mechanical strength, which may compromise its long-term performance due to retrogradation and environmental sensitivity.^3,4^ Therefore, the large-scale application of starch-based films and composites requires significant improvements in both water resistance and mechanical stability.

Reinforcing materials such as polymers, ceramics, and inorganic fillers have been investigated to enhance the internal structure of the starch matrix to address these challenges.^5^ These composites have shown improved tensile strength, reduced gas permeability, enhanced antimicrobial activity, and better resistance to radiation.^6^ For instance, semiconductor oxides (e.g., CuO, ZnO, Nb_2_O_5_, TiO_2_),^7–9^ with intermediate band gap values (2–4 eV), solubility under controlled pH, and water dispersibility, are promising candidates to overcome multiple drawbacks of starch films.^10,11^ Both bottom-up (e.g., precipitation, hydrothermal, ultrasonic, polymeric precursor routes) and top-down (e.g., milling) approaches can be employed to synthesize these oxides.^12–14^ Moreover, properties such as crystallographic phase, particle size and shape, distribution, and porosity can be finely tuned by controlling synthetic parameters like temperature, time, precursor source, and concentration.^15,16^ In this context, mining wastes, particularly waste rocks and tailings from ore beneficiation, represent a rich and underutilized source of metal precursors for nanomaterial synthesis.^17^ Their use reduces environmental liabilities by valorizing industrial byproducts while providing multifunctional reinforcement, enhancing mechanical strength, barrier performance, and UV shielding. Such advantages make these residues a promising sustainable alternative (low-cost and abundant) to conventional nanofillers. Besides that, their reuse could substantially reduce the environmental impacts associated with traditional tailings disposal, including air, water, and soil contamination.^18,19^

Manganese oxides and compounds are particularly attractive among transition metals due to their redox activity, wide availability, and versatility in synthesizing compounds. Manganese, a transition metal with multiple oxidation states (most commonly 2+, 3+, 4+, 6+, and 7+),^20^ is widely used in various applications, including batteries, glassmaking, ceramics, catalysis, and pigments. Manganese (IV) oxide, often in the γ-phase, is a known oxidizing agent in organic chemistry. At the same time, barium manganate (BaMnO_4_) serves as a milder oxidizer and is also used as a pigment and filler in the rubber and plastics industries.^21,22^ Furthermore, manganese is also an essential micronutrient for plants and animals. In plants, Mn concentrations typically range from 50 to 150 mg kg^–1^, with deficiency occurring below 10–50 μg g^–1^ dry weight.^23^ Moreover, the European Food Safety Authority (EFSA) has confirmed that manganese feed additives are among the least toxic essential elements and are considered safe up to 150 mg kg^–1^ in animal feed. At the low concentration used in this study (≤1 wt % of starch), manganese-based reinforcements can be regarded as eco-friendly.^24^

Recent studies have explored the valorization of manganese ore tailings, particularly from the Amazon region, as fillers to improve the mechanical performance of bituminous mixtures and to synthesize high-capacity conversion anodes for lithium-ion batteries.^25^ Manganese can be efficiently recovered from oxide-based tailings (extraction rates of ∼90%) via alkaline oxidative fusion, followed by conversion into nanostructured δ-MnO_2_ using green reductants such as ethanol or hydrogen peroxide.

In recent years, starch-based films have often been reinforced with synthetic oxides or untreated fillers, overlooking the potential of low-value mining byproducts. In contrast, our work introduces a simple yet powerful twist: we valorize manganese ore beneficiation residues (RBK), and their in situ-transformed phases, BaMnO_4_ and δ-MnO_2_, as dual-purpose reinforcing agents. Thus, our working hypothesis is that incorporating these residues into starch matrices will improve tensile strength and water vapor barrier performance and enhance UV-shielding efficiency, thereby promoting a circular economy approach by transforming mining waste into functional packaging materials. This approach not only delivers significant improvements in mechanical, barrier, and UV–Vis properties of starch films but also demonstrates a realistic, sustainable pathway to eco-friendly packaging, in line with the United Nations Sustainable Development Goals (SDGs), particularly SDG 9 (Industry, Innovation, and Infrastructure), SDG 12 (Responsible Consumption and Production), and SDG 13 (Climate Action). Beyond these scientific contributions, the proposed films may find a future perspective in applications such as biodegradable food packaging, protective coatings for light- and moisture-sensitive products, and short-life agricultural materials, providing a practical route for the valorization of mining residues into functional products.

## 2 Materials and Methods

### 2.1 Materials

Corn starch (Amidex 3001) was purchased from local suppliers. Urea (99%) and glycerol (99%) were obtained from Synth (Brazil). Manganese ore mining tailings were collected from the decommissioned Kalunga tailings dam, part of the Azul mining site operated by VALE S.A., located in the Carajás mining district (Pará, Brazil). Detailed information regarding the mineralogical and elemental composition of these tailings has been reported previously.^27^ Briefly, the material is characterized by high concentrations of kaolinite, quartz, and nonstoichiometric manganese oxides, including birnessite, todorokite, and nsutite. The average manganese content is approximately 10%, reflecting the specific beneficiation processes applied to manganese ore. Barium nitrate (99%) and potassium permanganate (KMnO_4_, 99%) were purchased from Carlo Erba (Italy).

### 2.2 Materials Synthesis from Mn Ore Tailings

#### 2.2.1 MnO_2_

Manganese recovery from tailings and subsequent synthesis of manganese oxide powders were carried out using a modified procedure based on a method previously reported by the authors.^26^ Briefly, alkaline fusion was performed at 250 °C for 3 h using a KOH-to-tailings mass ratio of 2:1. During the process, 10 mL of deionized water was added every hour to facilitate the reaction. After cooling, the fused solid was leached with a 3 M KOH solution, yielding a 0.04 M potassium manganese (K_2_MnO_4_) solution.

The relatively low manganese extraction efficiency (∼61% as K_2_MnO_4_) observed in this work can be attributed to the lower manganese content of the tailings, their significantly higher aluminum and silicon contents, and the use of a lower fusion temperature. For the synthesis of δ-MnO_2_, 100 mL of a 10 wt % hydrogen peroxide (H_2_O_2_) solution was added dropwise to 100 mL of the 0.04 M K_2_MnO_4_ solution under continuous stirring. The resulting suspension was stirred for 3 h, then filtered, washed with deionized water until neutral pH was achieved, and dried in an oven at 110 °C for 6 h.

#### 2.2.2 BaMnO_4_

A 0.1 M barium nitrate solution was added dropwise under vigorous stirring to the 0.04 M K_2_MnO_4_ stock solution until the characteristic green color disappeared, indicating the complete precipitation of BaMnO_4_. Excess barium ions were removed by washing the precipitate with deionized water until the pH reached ∼9 in order to prevent BaMnO_4_ disproportionation. Washing was continued until no BaSO_4_ was detected in the filtrate. The resulting solid was dried in an oven at 110 °C for 3 h. As a proof of concept, in order to minimize the formation of BaCO_3_ in the final BaMnO_4_ powders, an alternative synthesis route was tested by leaching the fusion product with a 1 M KOH solution to obtain a lower-concentration potassium manganate solution. The resulting BaMnO_4_ product contained less than 10% BaCO_3_. Nevertheless, only BaMnO_4_ synthesized from the 3 M KOH-derived K_2_MnO_4_ solution was used in the subsequent film reinforcement experiments.

### 2.3 Starch Composite Films

Starch films were prepared by using the casting method. Corn starch (5% w v^–1^) was initially dispersed in 250 mL of deionized water in a 600 mL beaker, along with a plasticizer mixture. A 1:1 (w w^–1^) urea/glycerol blend was employed as the plasticizer at a concentration of 30% (w w^–1^) relative to the starch content. The mixture was slowly homogenized using a stirring rod and heated to 80 °C.

Manganese-containing powders, RBK tailings, BaMnO_4_, and δ-MnO_2_, were added to the polymer solution at concentrations of 0.25, 0.5, and 1% (w w^–1^), under continuous stirring at 40 °C. Prior to addition, the powders were deagglomerated and dispersed in water by using an ultrasonic titanium horn (30% amplitude, 1 min). After 1 h of mixing, the suspension was allowed to cool naturally to approximately 40 °C to prevent bubble formation.

The resulting solution was cast into Teflon trays lined with silicone sheets and dried in a circulating-air oven at 40 °C for 24 h. A total of nine starch films were obtained and labeled as St/*x* *y*, where *x* indicates the type of filler (RBK, BaMnO_4_, or MnO_2_) and *y* represents the filler concentration (e.g., St/RBK 0.25).

### 2.4 Characterizations

Structural characterization was performed by X-ray diffraction (XRD) using a Shimadzu LabX XRD-6000 diffractometer with Cu Kα radiation (λ = 1.5406 Å). Scans were carried out in the 2θ range of 10–80°, at a scanning rate of 1° min^–1^. Fourier transform infrared spectroscopy (FTIR) was used to evaluate the vibrational modes of functional groups in the films and to assess possible structural modifications after filler incorporation. Spectra were recorded on a Bruker VERTEX FT-IR spectrometer with 32 scans, running between 4000 and 400 cm^–1^ spectral range, at 4 cm^–1^ resolution. Morphological analysis was conducted by using a scanning electron microscope (SEM, JEOL JSM-6510). Both the surface and cross sections of the starch films were examined. Samples were fractured in liquid nitrogen and subsequently coated with a thin carbon layer to improve the conductivity. Elemental composition and distribution were assessed on the same equipment by using an energy-dispersive spectroscopy (EDS) detector.

### 2.5 Water Vapor Permeability (WVP)

Water vapor permeability (WVP) was measured according to the ASTM E96 standard using the cup method. Briefly, film samples were cut into circular shapes and sealed over the opening of aluminum cups containing 6 mL of distilled water, ensuring no direct contact between the water surface and the film. Each test was performed in quintuplicate (*n* = 5).

The assembled cups were placed in a controlled-environment oven at 25 °C and 50% relative humidity. Weight loss was monitored over a 48 h period to determine the water vapor transmission rate. WVP was calculated according to eq 1:

1 $$\mathrm{WVP}\left(\mathrm{kg}\;h^{-1}\;m^{-1}\;Pa^{-1}\right)=\frac{w}{t}\frac{L}{A\Delta P}$$

In eq 1, *w*/*t* represents the slope of the weight loss curve (kg s^–1^), corresponding to the rate of mass loss over time, *L* is the average film thickness (m), *A* is the permeation area (m^2^), defined by the film’s exposed diameter, and Δ*P* (Pa) denotes the partial water vapor pressure difference between the inside and outside of the cup, determined by the relative humidity and temperature conditions of the assay. Accordingly, WVP is expressed in SI units as kilograms m^–1^ s^–1^ Pa^–1^.

### 2.6 Hydrophilicity/Hydrophobicity Degree (°)

Contact angles were measured by the sessile drop method using deionized water (3 μL droplets) to minimize artifacts from rapid absorption/spreading in hydrophilic films; the angle was recorded 1 s after deposition. For each formulation, drops at 3 distinct positions were measured, and results are reported as mean ± standard deviation.

### 2.7 Solubility by Mn-Leaching

The solubility of the oxide powders was evaluated by dispersing an appropriate mass in deionized water to obtain a final concentration of 50 mg L^–1^. Each sample was prepared in 50 mL of deionized water placed in Falcon tubes (50 mL), kept under static conditions for 24 h at room temperature and pH ≈ 6.7 (neutral). After incubation, the supernatant was separated by centrifugation (8000 rpm, 10 min, 25 °C) and subjected to instrumental analysis.

The release of manganese (Mn) was determined from films containing the oxides. Film specimens (∼20 mg; approximately 2 × 2 cm) were immersed in 10 mL of deionized water in 50 mL Falcon tubes and kept under static conditions for 24 h at room temperature.

Afterward, aliquots of the aqueous medium were collected for analysis. Manganese quantification was performed by flame atomic absorption spectrophotometry (FAAS) using a PerkinElmer PinAAcle 900T spectrometer. All measurements were carried out in triplicate, and results were expressed as the mean ± standard deviation.

### 2.8 Mechanical Test

Tensile strength measurements were carried out using a universal testing machine (EMIC DL2000), following the ASTM D638 standard. Tests were conducted at a crosshead speed of 5 mm min^–1^ using a 50 kgf load cell. Film specimens were conditioned prior to testing and evaluated at room temperature. At least ten replicates were tested for each formulation (*n* = 10).

### 2.9 Diffuse Reflectance Spectroscopy (DRS)

The ultraviolet–visible (UV–vis) radiation absorption capacity of the starch films was analyzed using a Shimadzu UV–vis spectrophotometer (model UV-2600) operating in reflectance mode over the 200–800 nm wavelength range. Reflectance data were converted into absorbance coefficient using the Kubelka–Munk function, which is commonly applied to diffuse reflectance measurements of solid samples.^28^ Additionally, the value is shown in energy unit (eV).

### 2.10 Transparency, Opacity, and UV Blocking

The optical properties of the starch-based films were determined from UV–vis absorbance spectra recorded in the range 200–800 nm using a spectrophotometer.

#### 2.10.1 Transparency

Film transparency at a given wavelength (λ) was calculated from the absorbance values (*A*) according to eq 2:

2 $$\%T\left(\lambda \right)=100\times 10^{-A\left(\lambda \right)}$$

where *A*(λ) is the absorbance at wavelength (nm). Transparency values were reported at 600 nm, corresponding to the visible range, which is commonly used as a reference point for film clarity.

#### 2.10.2 Opacity

Opacity of the starch-based films was determined from their optical transmittance at 600 nm. The transmittance spectra were recorded by using a UV–vis spectrophotometer, and the value at 600 nm (%T_600_) was used for calculations. Film thickness (mm) was measured with a digital micrometer at five random positions (mm), and the average value was used for normalization.

The opacity (mm^–1^) was calculated according to eq 3:

3 $$\mathrm{opacity}\left(A_{600}/d\right)=\frac{100-T_{600}}{\mathrm{thickness}\left(\mathrm{mm}\right)}$$

#### 2.10.3 UV Blocking

The UV-blocking efficiency was determined from the transmittance values, according to eq 4:

4 %UV−blocking(λ)=100−%T(λ)

where a global UV-blocking index was calculated as the average transmittance between 280 and 400 nm, and the integrated blocking percentage was expressed as:

### 2.11 Colorimeter

Color measurements were performed in CIE *L* × *a* × *b* × space using a Chroma Meter CR-410 (Konica Minolta) colorimeter. Initially, for the measurements, the instrument was calibrated with the white reference. For each formulation, triplicate readings were recorded at different positions. The starch film was taken as the baseline, and color differences were quantified as ΔE* (CIE76) relative to starch and whiteness index (WI), following eqs 5 and 6, respectively.

5 $$\Delta E=\sqrt{(L-L^{*}{)}^{2}+{\left(a-a^{*}\right)}^{2}+{\left(b-b^{*}\right)}^{2}}$$

6 $$\mathrm{WI}=100-\sqrt{(100-L{)}^{2}+a^{2}+b^{2}}$$

where L*, a*, and b* are the color coordinates of the starch control and *L*, *a*, and *b* are relative to the sample.

### 2.12 Statistical Analysis

Results are expressed as mean values ± standard deviation. Statistical analysis was carried out using one-way analysis of variance (ANOVA), followed by Tukey’s post hoc test to determine significant differences among sample means. A significance level of *p* < 0.05 was adopted in all cases. Data analysis was performed using Sisvar software.^29^

## 3 Results and Discussion

The morphology of the manganese-based materials used to reinforce the starch films was initially investigated by scanning electron microscopy (SEM), as shown in Figure 1. The images reveal clear differences in particle size distribution, shape, and homogeneity, which are expected to affect the degree of intercalation and interaction with the starch polymer matrix. The RBK sample (Figure 1a) consists of agglomerated particles with greater heterogeneity in size (243 ± 219 nm) and morphology and is dominated by randomly oriented plate-like structures. These platelets likely originate from kaolinite, which is one of the main mineral phases present in the tailings. BaMnO_4_ particles (Figure 1b) also display lamellar structures, with more homogeneous lateral size (399 ± 72 nm) and thickness (26 ± 6 nm). Additionally, BaMnO_4_ includes small particles and platelets with poorly defined morphology (107 ± 6 nm). In contrast, MnO_2_ obtained by direct reduction of potassium manganate with H_2_O_2_ forms nearly spherical nanoparticles with a mean diameter of 100 ± 22 nm. More generally, MnO_2_ can be synthesized by reducing permanganate precursors in the presence of agents, such as sugars, alcohols, or hydrogen peroxide.

**Figure 1:**
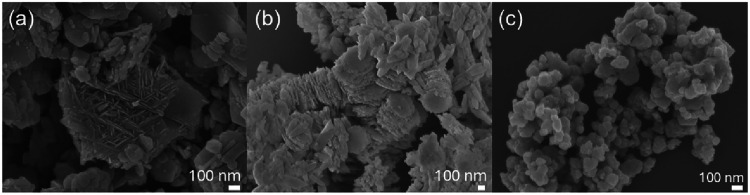
SEM image of manganese particles: (a) RBK, (b) BaMnO_4_, and (c) MnO_2_.

The X-ray diffraction patterns of starch-based films and reinforcing materials are presented in Figure 2. As expected, the presence of all three manganese-based fillers influenced the semicrystalline structure of the plasticized starch. Although partially masked by background noise, characteristic peaks at 14.2, 17.3, 20.1, and 22.3° were clearly observed for pure starch, consistent with what is reported in the literature.^30^

**Figure 2:**
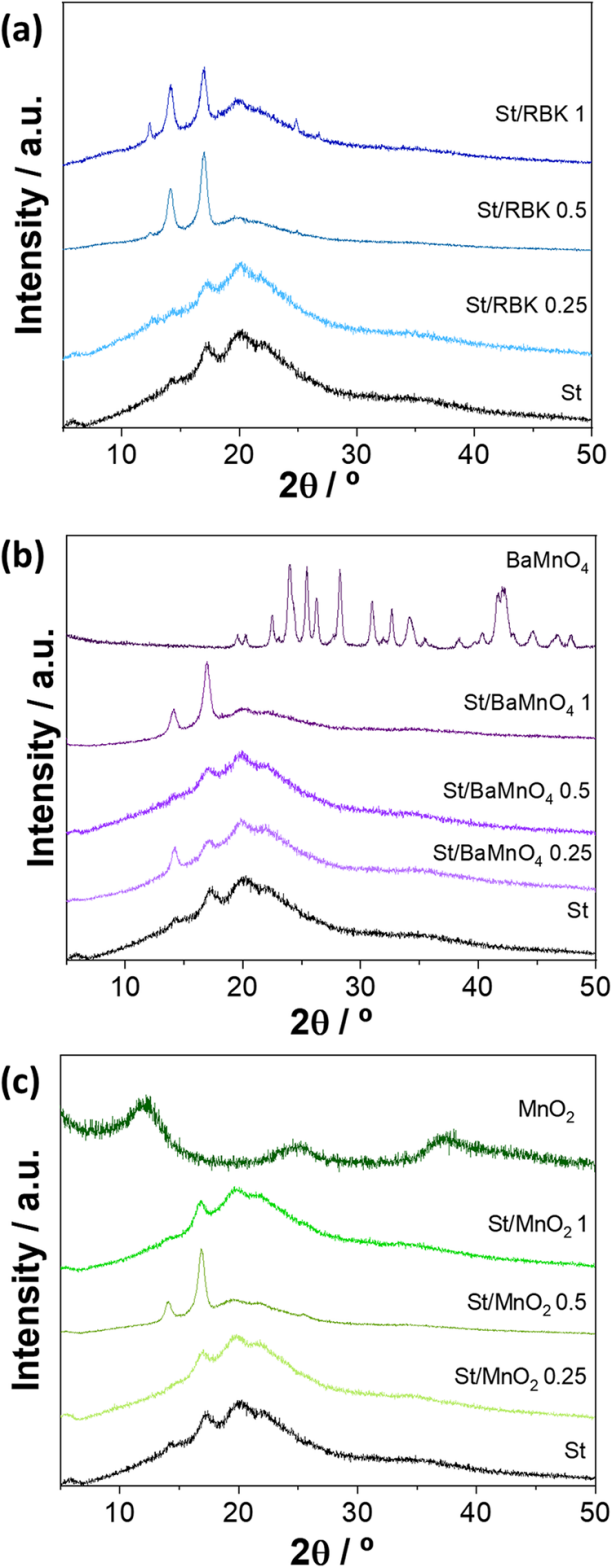
XRD diffractograms of composite films: (a) St/RBK, (b) St/BaMnO_4_, and (c) St/MnO_2_.

Despite the different nature of the reinforcing agents, increasing the filler content from 0.25 to 1% (w w^–1^) led to a noticeable enhancement in starch crystallinity, particularly reflected by intensified peaks at 14.2 and 15.9°. This behavior is likely due to the increased concentration and partial agglomeration of particles within the polymeric matrix, which may restrict the mobility of starch chains and promote a more organized structure.^31^

The crystalline phases of the reinforcing materials were also identified. In the case of BaMnO_4_ (Figure 2b), diffraction peaks matched both barium manganate (JCPDS No. 00-033-0164) and barium carbonate (BaCO_3_, JCPDS No. 05-0378). Rietveld refinement indicated a BaCO_3_-to-BaMnO_4_ ratio of approximately 2:1 (64% BaCO_3_, 36% BaMnO_4_), which is attributed to the highly alkaline conditions used during the synthesis. However, when BaMnO_4_ was precipitated from a potassium manganate solution derived from 1 M KOH, the BaCO_3_ content was reduced to below 10%. As previously described in the literature, the δ-MnO_2_ sample (Figure 2c) exhibited a poorly crystalline structure characteristic of potassium birnessite (JCPDS No. 42-1317).^32^ This material can be classified as a nanocrystalline form of δ-MnO_2_ with a layered structure capable of incorporating potassium ions and water. Rietveld analysis estimated an average crystallite size of approximately 4 nm. For the RBK sample at a 1% loading, a peak at 12.5° was observed. This signal corresponds to the interlayer spacing of kaolinite and may also include a residual contribution from birnessite originating from manganese ore in the tailings. In all cases, diffraction peaks corresponding to the reinforcing materials were not visible at concentrations below 1% (w w^–1^). This absence is likely due to the combination of low filler content and particle agglomeration within the dense polymer matrix, which hindered their detection by XRD.^33^ Furthermore, the nanocrystalline, poorly ordered nature of δ-MnO_2_ suppressed distinct diffraction peaks in the composites, which were observed only in the diffractogram of pure MnO_2_ powder.

FTIR spectra of neat starch and starch films reinforced with manganese-based residues (RBK, BaMnO_4_, and δ-MnO_2_) revealed two main regions of variation, as shown in Figure 3. First, the broad ν­(OH) stretching band (3500–3000 cm^–1^) showed a noticeable decrease in intensity upon filler incorporation. Second, changes were also detected in the 2000–1000 cm^–1^ region, associated with glycosidic C–O–C stretching and δOH bending modes. When comparing the different systems, RBK- and BaMnO_4_-based films presented the most pronounced spectral variations, with a progressive reduction of the ν­(OH) band and distinct modifications in the C–O–C region as the filler content increased. In contrast, δ-MnO_2_-reinforced films exhibited only minor differences relative to neat starch, maintaining a spectral profile closer to that of the control. In this case, the FTIR structural analysis demonstrates that RBK and BaMnO_4_ may promote stronger starch–filler interactions, as hydrogen bonding between hydroxyl groups of starch and the lamellar surfaces of the reinforcing agents, leading to reduced availability of free OH groups, affecting the glycosidic vibrations. In the case of δ-MnO_2_, the weaker spectral changes suggest limited chemical interaction, in agreement with previous FTIR reports where decreased O–H band intensity was attributed to hydrogen bonding between starch and added fillers.^34^

**Figure 3:**
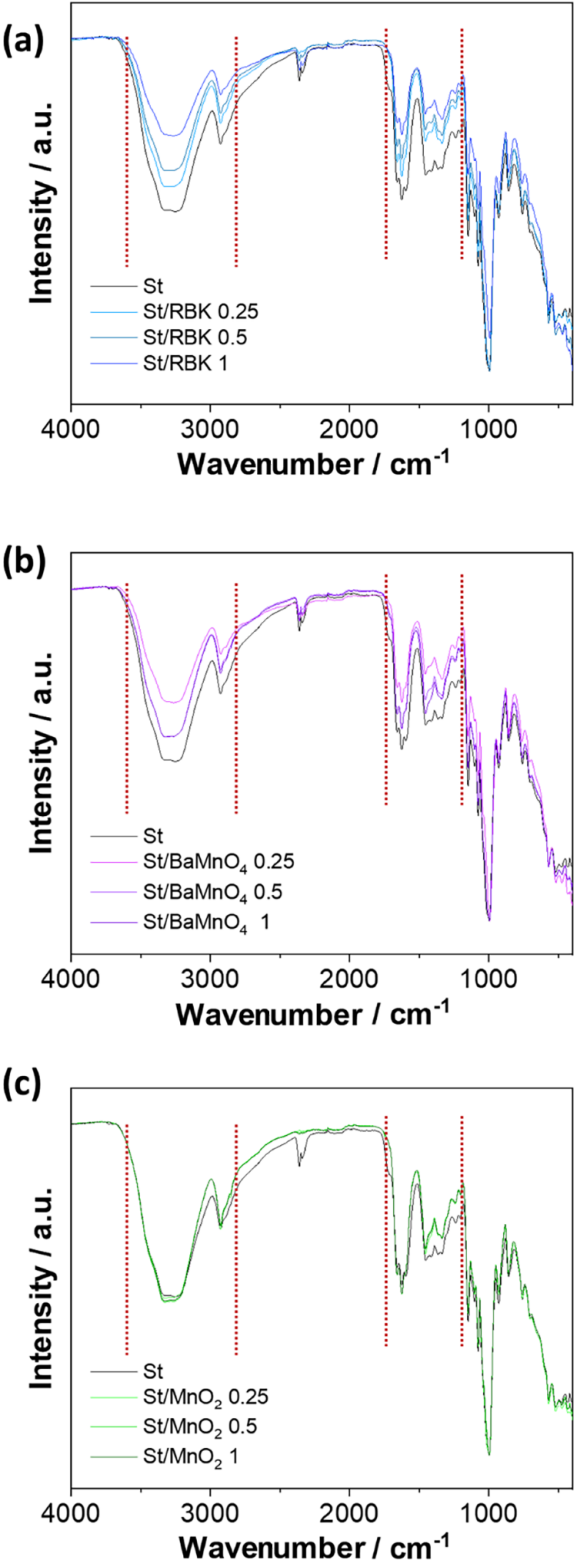
FTIR spectra of composite films: (a) St/RBK, (b) St/BaMnO_4_, and (c) St/MnO_2_.

Figure 4 shows SEM images of the surface of starch films reinforced with the three manganese-based materials. The analysis aimed to assess the presence and distribution of filler particles on the film surface, which play a key role in providing UV–vis blocking capabilities. RBK-based composites (Figure 4a–c) exhibit relatively smooth surfaces, possibly resulting from the alignment of clay particles at the top layer of the film. These particles, present in the micrometer size range, are consistent with the mineralogical composition of the tailings. Clay minerals have been previously reported to promote interaction with starch polymers due to their lamellar structure.^35^ However, only a few visible particles were detected on the film surface, suggesting a limited exposure of the filler. In the case of films containing BaMnO_4_ (Figure 4d–f), particles were more clearly observed on the surface, often forming agglomerates. These agglomerates appear as distinct domains, which may result from the simultaneous presence of both BaMnO_4_ and BaCO_3_. As expected, surface particle agglomeration tends to increase with increasing filler concentration. Agglomeration is even more pronounced in films reinforced with δ-MnO_2_, particularly at higher loadings. This behavior is likely attributed to the unique morphology of δ-MnO_2_, which forms through the coagulation of nanometer-sized particles into “honeycomb-like” structures. This morphology appears to hinder deep intercalation into the polymer matrix while promoting preferential surface localization and adhesion.^36^

**Figure 4:**
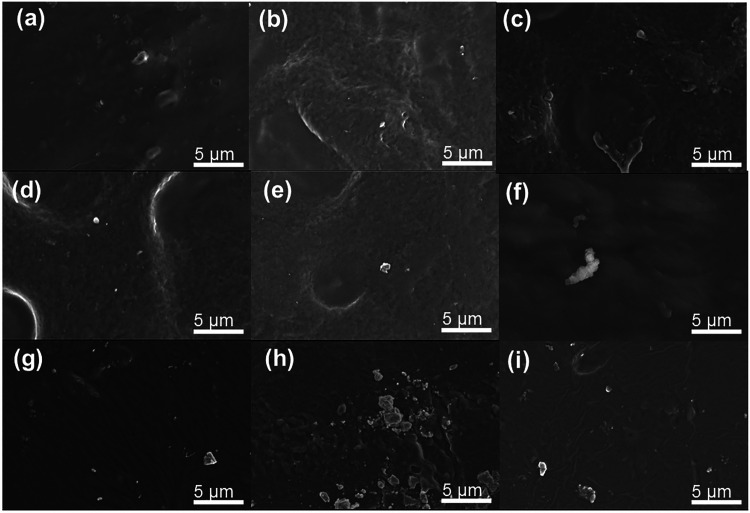
SEM microscopy image of starch films: (a) St/RBK 0.25, (b) St/RBK 0.5, (c) St/RBK 1, (d) St/BaMnO_4_ 0.25, (e) St/BaMnO_4_ 0.5, (f) St/BaMnO_4_ 1, (g) St/MnO_2_ 0.25, (h) St/MnO_2_ 0.5, and (i) St/MnO_2_ 1.

The presence of manganese-based reinforcing materials was further confirmed by energy-dispersive spectroscopy (EDS), as shown in Figure 5 for the 0.25% (w w^–1^) samples. As expected, the EDS spectrum of the RBK-reinforced film (Figure 4a) reflects the complex elemental composition of mining tailings derived from manganese ore beneficiation processes, such as crushing, grinding, scrubbing, size screening, jigging, and sink-and-float separation. In addition to manganese, elements such as aluminum, silicon, iron, potassium, magnesium, phosphorus, and sulfur were also detected. In contrast, the spectrum of the BaMnO_4_-reinforced film (Figure 5b) clearly shows the presence of barium. Interestingly, aluminum and silicon signals were significantly reduced or nearly absent, suggesting that the extraction of potassium manganate from the tailings was highly selective for manganese and did not coextract iron or other metal impurities. For the δ-MnO_2_-reinforced film (Figure 5c), only trace amounts of contaminants were observed, which are likely due to sample handling or the starch matrix itself. The potassium signal in the spectrum is consistent with the intrinsic structure of δ-MnO_2_, where potassium ions are intercalated between manganese oxide layers. Furthermore, oxygen evolution during the reduction of potassium manganate with hydrogen peroxide likely contributes to maintaining a clean oxide surface by minimizing the adsorption of foreign species. Importantly, manganese was clearly detected in all spectra, including those corresponding to the lowest filler concentration 0.25% (w w^–1^), confirming the effective incorporation of the reinforcing materials into the starch matrix.

**Figure 5:**
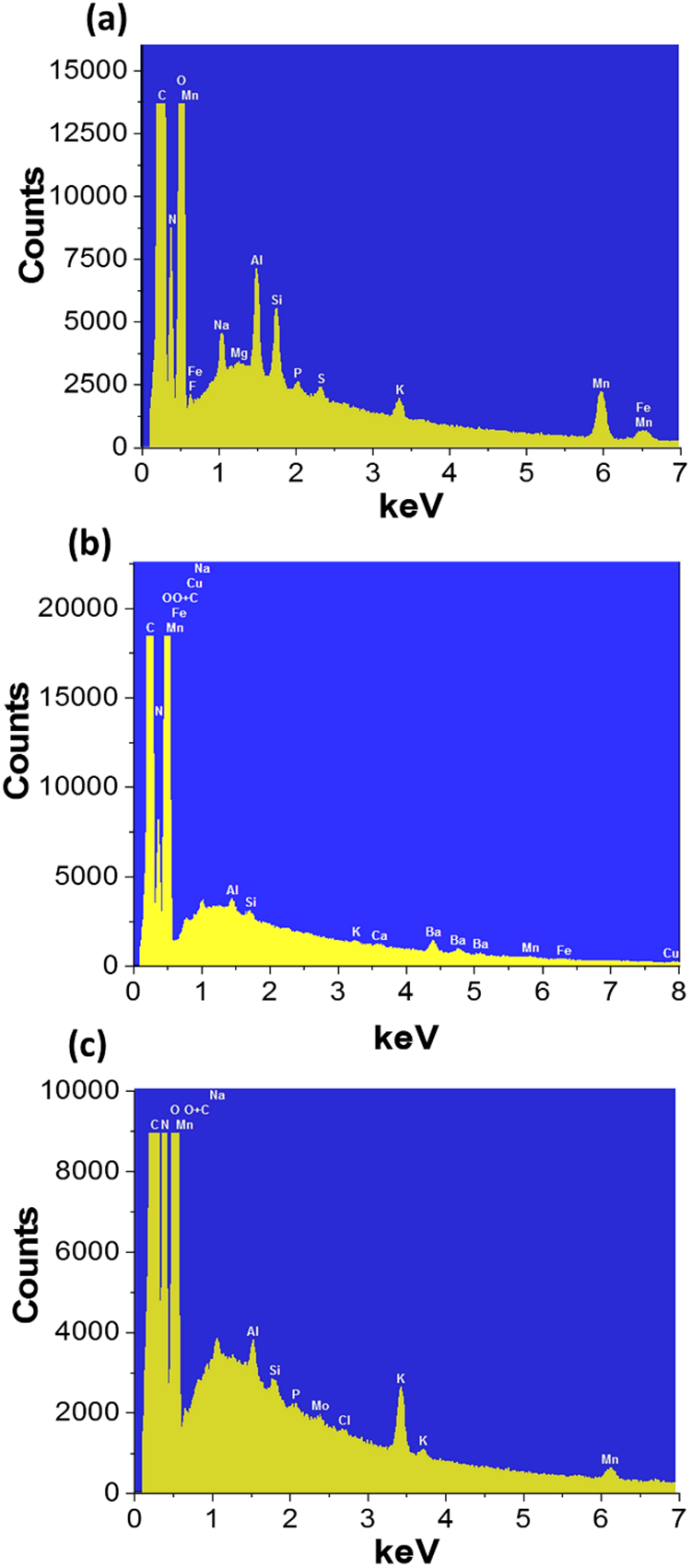
SEM-EDS element mapping of composite films: (a) St/RBK, (b) St/BaMnO_4_, and (c) St/MnO_2_ with 0.25% (w w^–1^).

Once the presence and distribution of manganese-based reinforcement materials in the starch films were confirmed, their effect on the functional packaging properties was evaluated. Figure 6 presents the water vapor permeability (WVP) results, demonstrating that the addition of RBK and BaMnO_4_ significantly enhanced the films’ barrier performance by reducing water vapor transmission. In contrast, films reinforced with δ-MnO_2_ showed increased WVP values compared to those of the pristine starch film, indicating a detrimental effect. Specifically, starch films exhibited a WVP of 4.9 ± 0.9 10^–10^ kg m^–1^ s^–1^ Pa^–1^. The lowest permeability values were recorded for St/RBK 0.5 (2.5 ± 0.5 × 10^–10^ kg m^–1^ s^–1^ Pa^–1^) and St/BaMnO_4_ 0.25 (2.8 ± 0.5 × 10^–10^ kg m^–1^ s^–1^ Pa^–1^). The incorporation of manganese-based fillers significantly affected the water vapor permeability (WVP) of the starch films (*p* < 0.05). As shown in Figure 5, different superscript letters indicate statistically significant differences according to one-way ANOVA followed by Tukey’s test. Films containing RBK and BaMnO_4_ exhibited similar permeabilities (letter b), while MnO_2_ films showed the highest values (letter c), similar to the starch film. This behavior is likely related to the surface arrangement and intrinsic properties of the filler. Manganese (III/IV) oxides are well-known for their high gas adsorption capacity, particularly moisture,^37,38^ which may compromise their effectiveness as water vapor barriers when incorporated into hydrophilic matrices. Furthermore, SEM images (Figure 3) showed a clear aggregation of δ-MnO_2_ on the film surface. Such surface-localized clusters may leave portions of the polymer matrix exposed and unprotected, contributing to higher vapor transmission rates. In contrast, the lamellar morphology of RBK and BaMnO_4_ (Figure 1a,b) appears to form a more compact and continuous structure within the matrix, which impedes water vapor diffusion. This barrier effect is consistent with previous findings on clay-like materials, where gas diffusion across lamellar systems is significantly restricted due to the tortuous path created by overlapping platelets.^39,40^

**Figure 6:**
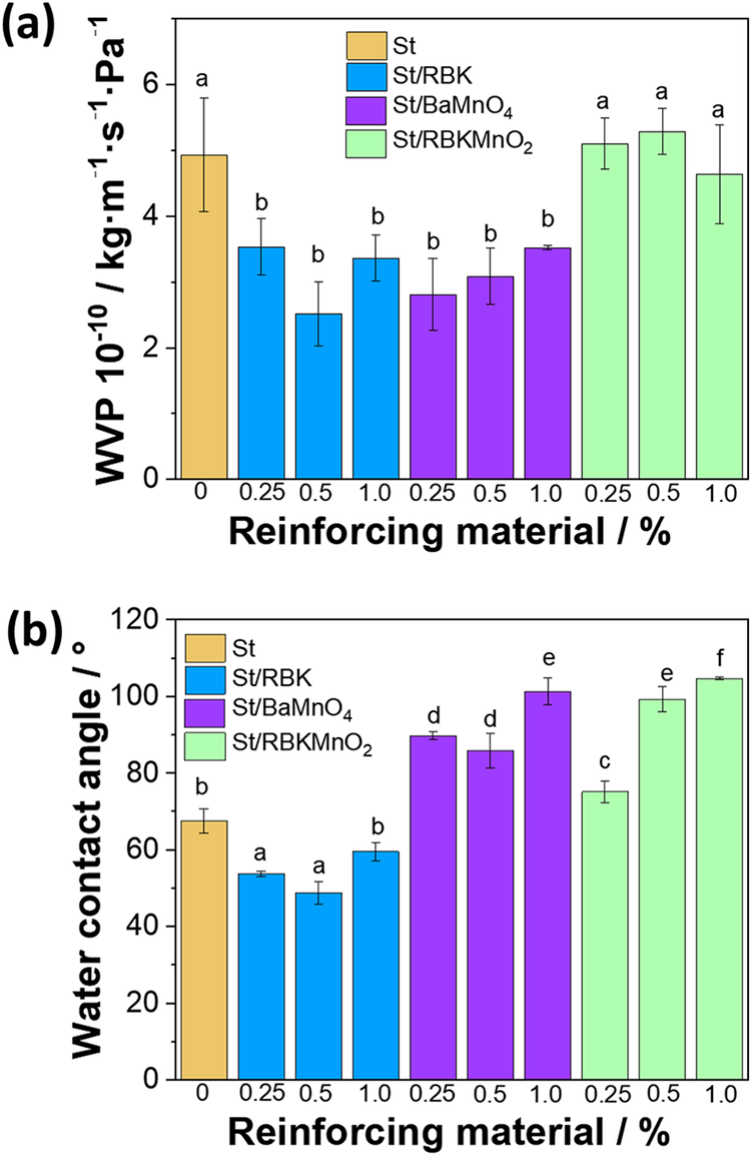
(a) Water vapor permeability (WVP) and (b) contact angle (hydrophilicity/hydrophobicity degree, °) of starch films reinforced with RBK, BaMnO_4_, and MnO_2_ residues. Values are expressed as mean ± standard deviation. Different superscript letters indicate statistically significant sample differences (one-way ANOVA, Tukey’s test, *p* < 0.05).

The contact angle measurements (Figure 6b) revealed distinct wettability trends depending on the type of reinforcement. Neat starch films exhibited an intermediate wettability (67.6 ± 3.2°, group b), while RBK incorporation significantly reduced the values to 53.8 ± 0.7 and 48.7 ± 2.9° for 0.25 and 0.5% loadings, respectively (group a), indicating enhanced hydrophilicity. This behavior can be attributed to the lamellar and silicate-rich nature of RBK, which increases the density of surface hydroxyl groups and polar sites available for hydrogen bonding with water. A similar effect was reported for starch/MMT system, where the exposure of hydrophilic clay surfaces promoted higher water spreading on the composite films.^41^

In contrast, St/BaMnO_4_-and St/MnO films exhibited superior contact angles, progressively reaching 89.7 ± 1.0° (BaMnO_4_ 0.25%, group d), 100.1 ± 3.6° (BaMnO_4_ 1%, group e), and 104.9 ± 0.3° (MnO_2_ 1%, group f), clearly surpassing 90°, evidencing a transition to hydrophobic surfaces. These increases were statistically significant compared to starch and RBK groups (*p* < 0.05, Tukey’s test). Such behavior may arise from the lower density of surface hydroxyl groups and the preferential localization of these oxides as aggregated domains at the film surface, which decreases surface energy and promotes water repellency.^42^ These wettability trends are consistent with the WVP results. RBK-based films, although more hydrophilic, showed reduced vapor permeability (down to 2.5 ± 0.5 × 10^–10^ kg m^–1^ s^–1^ Pa^–1^ for St/RBK 0.5) due to the tortuous diffusion pathway created by lamellar particles. In the case of BaMnO_4_, it not only improved barrier performance (as low as 2.8 ± 0.5 × 10^–10^ kg m^–1^ s^–1^ Pa^–1^ for St/BaMnO_4_ 0.25) through lamellar packing but also enhanced surface hydrophobicity, further limiting water uptake. Meanwhile, St/MnO_2_ films, despite displaying strong hydrophobicity (≥100°), exhibited higher WVP values, likely due to particle aggregation at the surface observed by SEM, promoting unprotected regions of the polymer matrix exposed to vapor diffusion. Together, these findings highlight that wettability and barrier performance are not necessarily linearly correlated, but rather depend on the interplay between surface chemistry, morphology, and filler distribution.

The solubility of films was evidenced by Mn-release from nanocomposite materials.

After 24 h in water (20 mg film in 10 mL), the films released measurable amounts of Mn; The results evidenced that Mn was available to external medium following MnO_2_ > BaMnO_4_ ≫ RBK. St/MnO_2_ films showed the highest 0.61 ± 0.13 mg L^–1^ at 0.25%, 1.89 ± 0.18 mg L^–1^ at 0.5%, and 3.06 ± 0.10 mg L^–1^ at 1%, which corresponds to 0.30, 0.95, and 1.53 mg g^–1^ film. BaMnO_4_ release at lower levels, 0.42 ± 0.01, 0.88 ± 0.04, and 1.39 ± 0.03 mg L^–1^ (≈0.21–0.70 mg g^–1^). RBK remained near the analytical floor (∼0.02–0.04 mg L^–1^, ≤0.02 mg g^–1^), indicating negligible Mn solubilization from this mineral residue.

This result corroborates the particle solubilization and manganese amount in the fillers (using Mn mass fractions: MnO_2_ = 0.632; BaMnO_4_ = 0.214), the films released only a fraction of what is available: ∼19–30% for MnO_2_ (0.25–1%) and ∼32–41% for BaMnO_4_. Thus, the starch matrix hydrates and opens interfaces, allowing partial solubilization/dispersion of Mn species without disintegrating the film. Moreover, Mn particles initially at 50 mg L^–1^ confirm the intrinsic solubility contrast: MnO_2_ powder released 7.86 ± 0.01 mg L^–1^ Mn (≈25% of its theoretical limit), whereas BaMnO_4_ powder gave only 0.53 ± 0.14 mg L^–1^ (≈5%). The same behavior appears in the films, and the higher absolute release for MnO_2_ 1% is consistent with its higher Mn content and purity and better interfacial accessibility in water. In a simple way, water uptake softens the starch network enough to liberate Mn species, especially from MnO_2_-filled films, while RBK contributes practically no soluble Mn.

The tensile strength of all starch-based films was evaluated, with the results presented in Figure 7 and Table 1. Compared to the pristine starch film, which exhibited a tensile strength of 3.5  ±  0.7 MPa, significant improvements were observed in composites reinforced with RBK and BaMnO_4_. The tensile strength increased to 14  ±  2 MPa for St/RBK 0.5 and reached 20  ±  4 MPa for St/BaMnO_4_ 0.5. In contrast, MnO_2_-reinforced films did not show comparable enhancement, with a maximum value of 7  ±  3 MPa observed for St/MnO_2_ 1. The mechanical properties of the starch films were also significantly influenced by the addition of manganese-based fillers (*p* < 0.05). Different superscript letters in Table 1 indicate statistically significant differences according to one-way ANOVA followed by Tukey’s test. For tensile strength, BaMnO_4_- and RBK-reinforced films exhibited significantly higher values compared to neat starch (letter a), whereas MnO_2_-containing films showed lower strength (letter c). In contrast, elongation at break (strain) decreased sharply with the incorporation of RBK and BaMnO_4_ (letters b and c), while MnO_2_-based films maintained the highest flexibility among the reinforced systems, although still distinct from the neat starch control.

**Figure 7:**
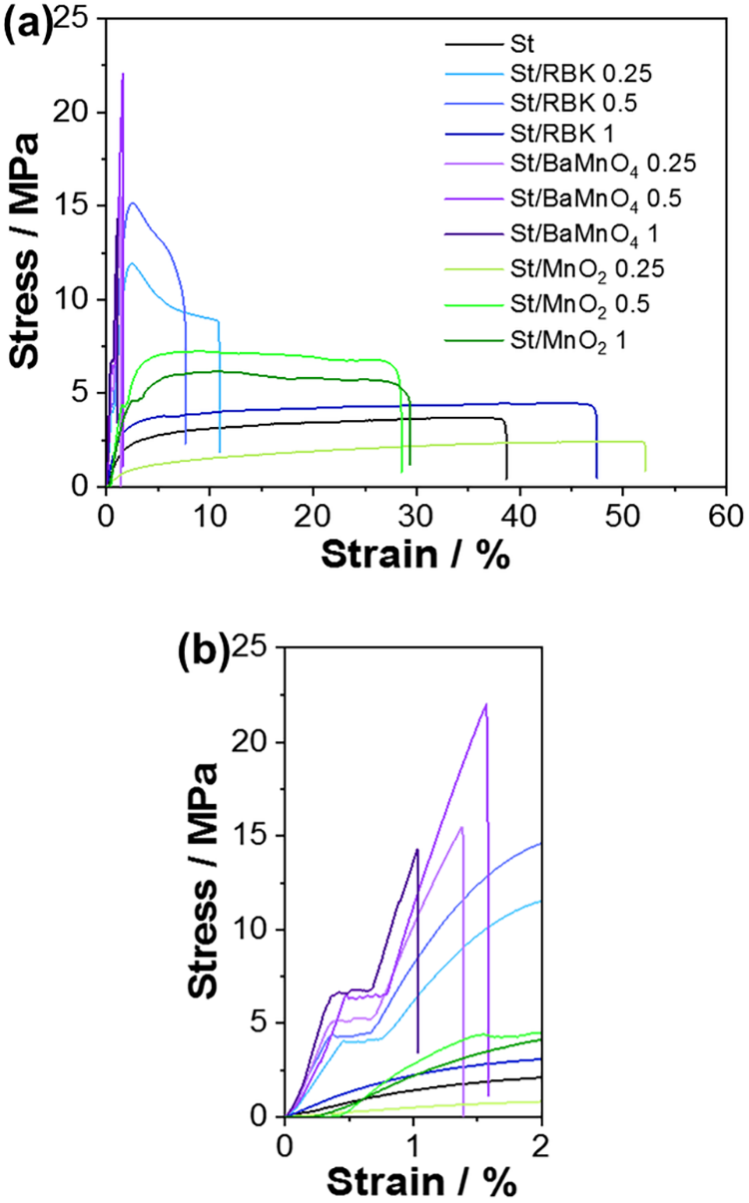
Mechanical traction of composite films (a) with a magnified view of the lowest strain region (b).

**Table 1:** Mechanical Properties of Starch Films Reinforced with RBK, BaMnO_4_, and MnO_2_ Residues^a^

**samples**	**stress/MPa**	**strain/%**
St	3.5 ± 0.7^a^	34 ± 7^b^
St/RBK 0.25	12 ± 5^b^	10 ± 8^a^
St/RBK 0.5	14 ± 2^b^	5 ± 1^a^
St/RBK 1	5 ± 2^a^	33 ± 15^b^
St/BaMnO_4_ 0.25	17 ± 3^b^	1.3 ± 0.2^a^
St/BaMnO_4_ 0.5	20 ± 4^c^	1.4 ± 0.2^a^
St/BaMnO_4_ 1	15 ± 6^b^	1.2 ± 0.2^a^
aSt/MnO_2_ 0.25	2 ± 1^a^	53 ± 7^c^
St/MnO_2_ 0.5	7 ± 2^a^	20 ± 14^b^
St/MnO_2_ 1	7 ± 3^b^	23 ± 11^b^

a Results are expressed as mean ± standard deviation. Different superscript letters within the same column indicate statistically significant differences among samples (one-way ANOVA, Tukey’s test, *p* < 0.05), Sisvar software.

These findings are consistent with the WVP results, showing that RBK and BaMnO_4_ are the most effective reinforcement agents among those tested. The improved mechanical performance is attributed to the intercalation of the reinforcing particles within the starch matrix, which facilitates stress transfer from the polymer to the filler, reduces stress concentration points, and delays crack propagation. Additionally, the anisotropic nature of lamellar particles such as RBK and BaMnO_4_ allows them to align preferentially under stress, contributing to strain dissipation along the direction of tensile load.^43–45^ This structural arrangement provides a cushioning effect, enhancing mechanical resistance in the direction of the applied force. No significant dependence of tensile strength on filler concentration was observed across the studied range, indicating that even at low loadings, the reinforcement effect remains for RBK and BaMnO_4_. However, the tensile strength values exhibited relatively high standard deviations, which became more evident at the highest concentration (1%). This result indicates that manganese reinforcements were heterogeneously distributed within the polymeric matrix, creating potential rupture points. Such behavior is likely related to particle agglomeration, which explains why the best mechanical performance was achieved at lower concentrations (St/RBK and St/BaMnO_4_).

The UV–vis radiation barrier properties of the composite films were evaluated through diffuse reflectance spectroscopy (DRS). As expected, the incorporation of manganese-containing materials (Figure 8b–d) introduced new absorption bands into the UV–vis spectrum of the starch films. The RBK residue exhibited a broad and intense absorption band centered around 266 nm. This observation reflects the presence of transition metal oxides, particularly manganese and iron oxides, in the tailings, whose characteristic absorption typically falls within this spectral region.^46^ Interestingly, the optical absorption from RBK particles was most pronounced at the lowest filler concentration (0.25% w w^–1^). This behavior may be explained by the tendency of higher concentrations to promote particle aggregation, which reduces the light scattering due to limited interfacial contact between the filler and the polymer matrix.

**Figure 8:**
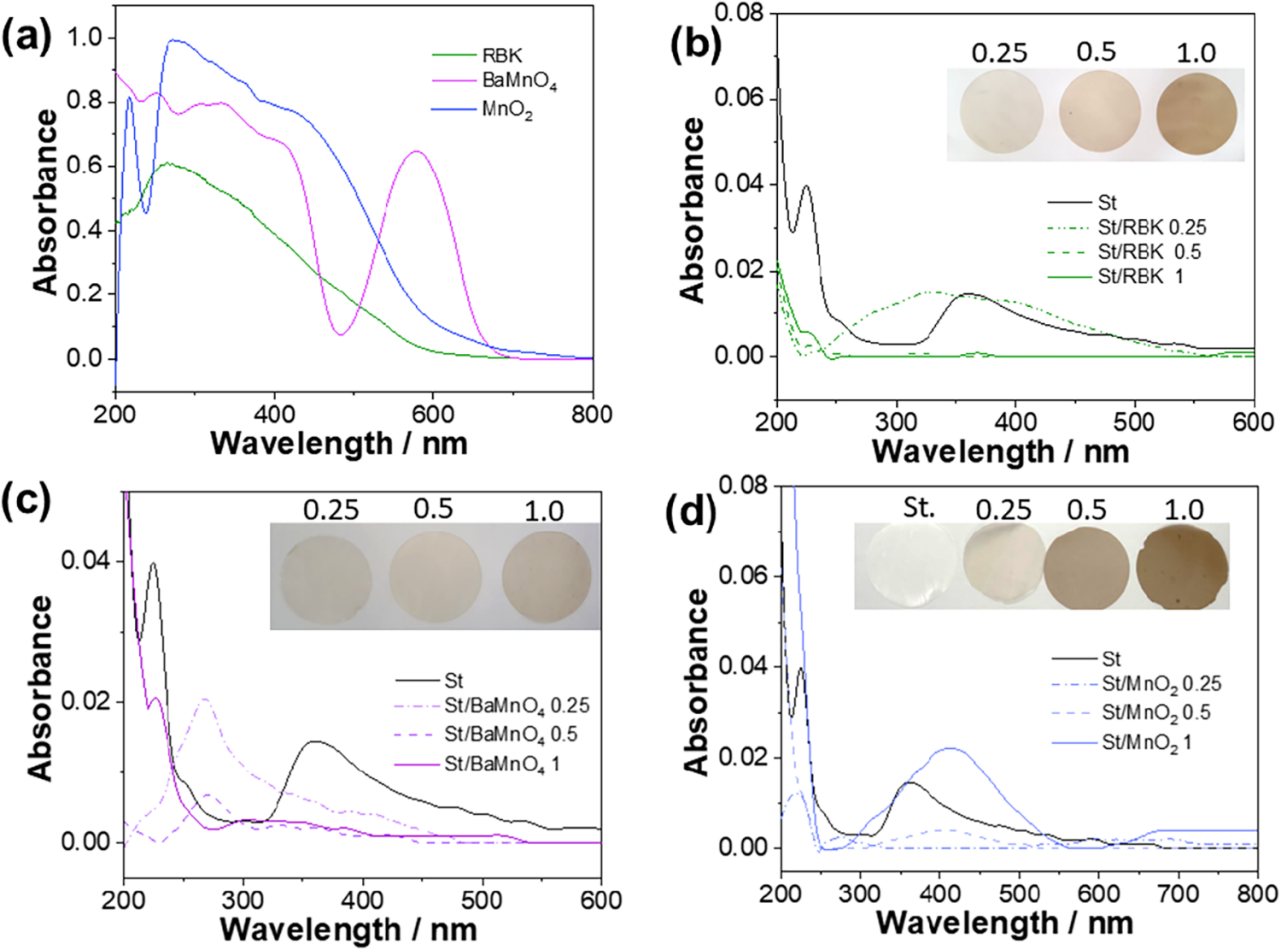
UV–vis spectrum of (a) Mn ore byproducts, (b) St/RBK, (c) St/BaMnO_4_, and (d) St/MnO_2_ films. “Photographs provided by the authors. Copyright 2025.”.

BaMnO_4_, a dark blue powder, exhibited a distinct absorption feature near 600 nm (Figure 8a), which was attributed to the presence of the manganate ion (MnO_4_^2–^). In this species, Mn^6+^ is tetrahedrally coordinated, and the 600 nm band corresponds to ligand-to-metal charge transfer (LMCT) transitions.^47^ Additionally, a minor absorption band was observed near 200 nm, likely arising from BaCO_3_, which has a wide band gap (Eg ≈ 5.5 eV). Surprisingly, the composite films containing BaMnO_4_ (Figure 8) did not display the characteristic 600 nm band, regardless of the filler concentration. Instead, a new absorption band appeared around 267 nm. Furthermore, the St/BaMnO_4_ films exhibited a brownish-gray color rather than the expected blue, suggesting that a chemical transformation may have occurred during film preparation. It is hypothesized that urea, used as a plasticizer in the film formulation, may have contributed to the reduction of BaMnO_4_ to lower oxidation states of manganese. Film processing conditions (40–80 °C for 1 h) are compatible with the thermal decomposition of urea, leading to ammonia release and a reducing environment. Such a behavior could favor the partial conversion of BaMnO_4_ to MnO_2_ or Mn_2_O_3_. The appearance of a broad absorption band with a maximum peak at 267 nm and extending up to 500 nm suggests the presence of multiple overlapping electronic transitions, likely associated with different manganese oxidation states. This spectral feature may reflect the partial transformation of BaMnO_4_ into oxides such as MnO_2_ and Mn_2_O_3_. These phases, containing Mn^4+^ and Mn^3+^ respectively, are known to exhibit ligand-to-metal charge transfer (LMCT) and d–d transitions, which can contribute to wide absorption profiles in the UV–vis region. Structural disorder, oxygen vacancies, or phase mixtures may further broaden absorption.^48^ Additionally, during film preparation, the polymeric solution gradually darkened after BaMnO_4_ was added, providing visual evidence of manganese reduction and the formation of the Mn­(IV/III/II) species.

Figure 8d presents the UV–vis spectra of starch films reinforced with δ-MnO_2_. A distinct peak at 413 nm (observed in St/MnO_2_ 1) is attributed to ligand-to-metal charge transfer (LMCT) transitions.^49^ The original δ-MnO_2_ powder displayed an absorption band near 320 nm, consistent with the nanostructured nature of potassium birnessite synthesized from mining residues.^50^ However, this nanometric signature is no longer visible in the spectrum of the composite film, likely due to particle agglomeration during film formation. As previously discussed, RBK and BaMnO_4_ composites demonstrated the most significant impact on the UV–vis barrier properties. This performance difference can be linked to the morphology of the reinforcing particles. Lamellar structures such as those found in RBK and BaMnO_4_ are more likely to align between polymer chains, thereby enhancing light-blocking efficiency through increased surface coverage and optical path interference.^51^ Therefore, it can be concluded that the pristine RBK mining residue provides more effective reinforcement compared with its derivatives (BaMnO_4_ and MnO_2_), particularly in terms of improving the optical barrier performance of starch-based films.

The optical band gap of the films was estimated from Tauc plots (Figure 9). This parameter is relevant because it reflects the electronic structure and the ability of the films to absorb or block UV–visible radiation, which is directly related to potential applications in packaging and protective coatings. Neat starch displayed two apparent band gaps (∼2.7 and ∼4.6 eV), a feature attributed to its semicrystalline nature, where amorphous domains generate localized states while crystalline regions preserve the intrinsic wide gap.^52^ For mining residue films (RBK, Figure 9b), low loadings (0.25–0.5 wt %) showed higher apparent gaps (5.2–5.8 eV), dominated by wide-gap silicate phases. At 1 wt %, particle agglomeration/percolation enhanced disorder and scattering, reintroducing the starch-related onset around 2.5 eV. In BaMnO_4_-derived films (Figure 9c), the starch-related 2.6 eV edge disappeared, while the main absorption progressively red-shifted from 4.8 eV (0.25 wt %) to 3.7 eV (1 wt %). This behavior may be associated with the conversion of BaMnO_4_ into MnO_2_/MnOx during processing, as indicated by the color transition from blue to brown, consistent with typical redox interconversions reported for manganese compounds.^53^ In MnO_2_ films, low concentrations suppressed the ∼2.6 eV onset, showing only the high-energy edge (∼5.2 eV), whereas at 1 wt %, a low-energy edge (∼2.3 eV) reappeared, indicating agglomeration-induced disorder and localized states. Overall, the fillers led to distinct optical responses: neat starch showed dual gaps, BaMnO_4_-derived films exhibited progressive red-shift due to oxidation, MnO_2_ revealed subgap states at higher loadings, and RBK resembled this behavior with the starch-related onset reappearing at 1 wt %. Such trends are consistent with literature reports that increasing nanoparticle loading can induce new sub-band gaps through percolation and interparticle interactions.^54^

**Figure 9:**
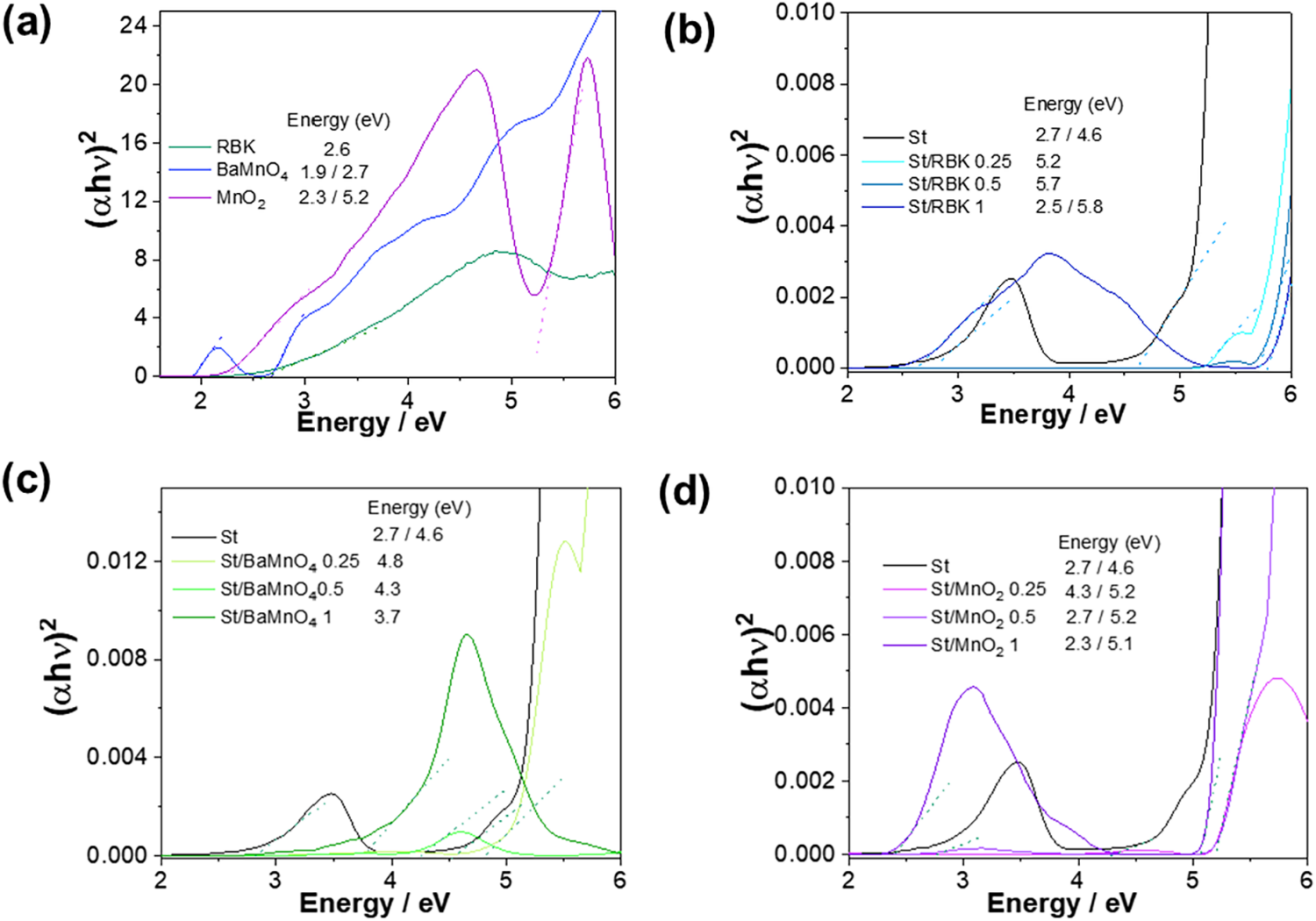
Tauc plots (α*h*ν)*n*(\α*h*\nu)∧*n*(α*h*ν)*n* versus photon energy (*h*ν)­(*h*\nu)­(*h*ν) for pure powders (a), starch-based films reinforced with RBK (b), BaMnO_4_ (c), and MnO_2_ (d). The dashed lines indicate the linear regions used for extrapolation of the optical direct band gap (Eg).

This result is accomplished with the color change in the composite films shown in Table 2. Colorimetry revealed filler-dependent changes in film appearance. Lightness (L*) decreased with increasing loading (one-way ANOVA, *p* < 0.05), with the strongest darkening for MnO_2_ and RBK, while BaMnO_4_ remained close to neat starch at ≤0.5 wt % and showed a clearer drop only at 1 wt %. The a* parameter increased (reddish shift) with loading, significantly across all RBK contents, for MnO_2_ at ≥0.5 wt %, and for BaMnO_4_ at 1 wt %, whereas b* increased in all systems, indicating progressive yellowing.

**Table 2:** Color Parameters (CIE L*a*b*) of Starch-Based Films^a^

**sample**	L*	a*	b*	ΔE* vs starch	**WI**	**opacity/mm** ^**–1**^
St	91.20 ± 0.30^a^	1.26 ± 0.33^a^	–2.03 ± 1.35^a^	0.00 ± 0.00^a^	90.81 ± 0.15^f^	63.7 ± 0.6^a^
St/RBK 025	81.89 ± 1.25^b^	2.31 ± 0.20^b^	5.75 ± 1.18^e^	2.92 ± 0.35^a^	89.53 ± 0.22^f^	104.8 ± 3.1^b^
St/RBK 05	79.59 ± 1.13^d^	2.73 ± 0.52^b^	7.75 ± 0.09^e^	3.87 ± 0.51^b^	89.36 ± 0.45^e^	172.9 ± 5.1^c^
St/RBK 1	66.17 ± 1.23^f^	5.11 ± 0.25^d^	16.22 ± 0.72^f^	15.88 ± 1.03^d^	79.06 ± 0.91^d^	246.3 ± 9.0^d^
St/BaMnO_4_ 025	89.62 ± 0.20^a^	1.25 ± 0.06^a^	0.42 ± 0.32^b^	6.46 ± 1.06^b^	86.40 ± 0.84^e^	103.9 ± 4.2^b^
St/BaMnO_4_ 0.5	89.56 ± 0.41^a^	1.38 ± 0.07^a^	1.46 ± 0.37^b^	17.72 ± 1.45^d^	75.88 ± 1.41^c^	107.0 ± 4.0^b^
St/BaMnO_4_ 1	82.48 ± 0.50^b^	2.54 ± 0.07^b^	11.18 ± 0.91^c^	35.97 ± 1.72^f^	57.62 ± 1.76^a^	109.5 ± 3.2^b^
St/MnO_2_ 025	86.79 ± 0.70^c^	1.77 ± 0.06^a^	2.65 ± 0.80^b^	12.18 ± 1.72^c^	80.85 ± 1.56^d^	81.4 ± 3.3^a^
St/MnO_2_ 05	78.19 ± 1.12^d^	2.89 ± 0.19^b^	9.89 ± 0.90^c^	15.27 ± 0.85^c^	77.99 ± 1.00^c^	118.2 ± 2.7^b^
St/MnO_2_ 1	63.01 ± 1.58^e^	5.92 ± 0.32^c^	19.81 ± 0.73^d^	31.22 ± 1.44^e^	62.14 ± 1.44^b^	254.9 ± 0.6^d^

a Values are mean ± SD (*n* ≥ 3). Letters (superscript) indicate significant differences among composites (Tukey’s HSD, α = 0.05). Baseline for ΔE*: Starch.

The total color difference (ΔE*) relative to the starch control followed the order MnO_2_ > RBK > BaMnO_4_. Even the lowest RBK and MnO_2_ concentration resulted in perceptible color changes (ΔE* above the usual visibility threshold), whereas BaMnO_4_ remained near that threshold up to 0.5 wt % and increased more sharply at 1 wt %. This result is relative to the intrinsic absorption of the fillers and the greater optical path tortuosity as particle content increases. The whiteness index (WI) declined in parallel with L* and the rises in a* and b*, again with a milder decrease for BaMnO_4_ at ≤0.5 wt % and a more pronounced reduction for RBK and MnO_2_. Thus, BaMnO_4_ at low loadings offers minimal visual impact while still enabling functional gains, whereas MnO_2_ affords the largest optical attenuation at the expense of stronger coloration (RBK shows intermediate behavior). This color change in the composite films suggests that broadband absorption by the fillers and light-scattering-induced path lengthening act synergistically, explaining both the visible tint and the enhanced UV shielding, as also observed for MnO_2_-based films that completely block UV above ∼1 wt %.^55^

The opacity results at 600 nm (Table 3) showed differences among the starch films reinforced with distinct fillers. The neat starch film exhibited the lowest opacity (63.7 ± 0.6 mm^–1^, group a), consistent with its relatively transparent appearance. Incorporation of RBK residues significantly increased opacity in a concentration-dependent manner from 104.8 ± 3.1 mm^–1^ at 0.25% to 172.9 ± 5.1 mm^–1^ and up to 246.3 ± 9.0 mm^–1^ at 1%. This strong turbidity effect reflects the heterogeneous mineral composition of RBK, which promotes intense light scattering and decreased transmittance.^56^ In contrast, St/BaMnO_4_ films maintained similar opacity values across all concentrations (103.9–109.5 mm^–1^). This trend suggests that BaMnO_4_ particles, even at higher loadings (1%), interacted more homogeneously with the polymeric network, avoiding a drastic loss of transparency.

**Table 3:** Comparative Performance of Starch-Based Films Reinforced with Conventional Nanofillers from the Literature and with Mn Residues (This Work)

**starch matrix**	**reinforcement (max w w** ^**–1**^ **%)**	**tensile strength (MPa)**	**WVP**	**optical/UV–vis**	**references**
cassava starch	1% nanocellulose (CNF)	5.14 to 25.58	n.d.	5.84% variation of transparence	^59^
cationic starch	3 MMT/ 1% ZnO	∼3 to 5.42	8.75 10^–7^ to 3.04 × 10^–7^ g m^–1^ h^–1^ Pa^–1^	transmittance decreased from 83.11 to 7.05 (600 nm)	^60^
potato starch	nano-SiO_2_ 0.3 (w v^–1^) 100 nm	∼15 to ∼25	The rate decreased from ∼880 to 789.41 g m^–2^ d^–1^	transmittance decreased from ∼60 to ∼30% (600 nm)	^61^
corn starch	4% montmorillonite (MMT)	2.5 to 5 (Na-MMT) and 6.2 (Ag-MMT)	7.4 10^–7^ G m^–1^ h^–1^ Pa^–1^ to 4 (Ag-MMT) and ∼6 (Na-MMT)	absorbance peak 360–745 nm (Ag-MMT)	^62^
corn starch	5.7% zeolite A (8 g/140 g)	∼0.3 to 0.8	n.d.	opacity increased from 0.65 to 0.74	^63^
potato starch	0.5–2% graphene	1 to 1.68 (1%)	n.d.	decreased the transparency UV–vis (200–800 nm) from 6 to 2% (1%)	^64^
cassava starch	2% CuO/k-carrageenan	26.19 to 101.67	1.17 10^–10^ to 1.59 10^–10^ g m^–1^·s^–1^·Pa^–1^	opacity increased from 2.18 to 7.2 (2%)	^65^
corn starch	0.25 – 1% Mn minings (RBK, BaMnO_4_, and MnO_2_)	3.5 to 20 (St/BaMnO_4_ 0.5)	4.9 10^–10^ to 2.5 10^–10^ St/RBK 0.5 and 2.8 10^–10^ kg·m^–1^·s^–1^·Pa^–1^ St/BaMnO_4_ 0.25	opacity at 600 nm increased significantly from 63.7 mm^–1^ to 254.9 mm^–1^ (St/RBK 1) and 245.3 (St/MnO_2_ 1)	this work

For MnO_2_-based films, at low content (0.25%), the opacity remained low (81.4 ± 3.3 mm^–1^, a), while 0.5% resulted in a moderate increase (118.2 ± 2.7 mm^–1^). However, the highest concentration (1%) led to a sharp rise (254.9 ± 0.6 mm^–1^), the highest one. This quick change may be relative to particle agglomeration and higher refractive index mismatch at elevated MnO_2_ loadings, which considerably enhanced light scattering.^57^

In Figure 10, it is possible to verify that the filler concentration promoted an increase in UVB > UVA blocking. The neat starch film (UVB = 45.96 ± 1.38%; UVA = 24.61 ± 0.56%; Total UV = 30.78 ± 0.80%). St/RBK 1 and St/MnO_2_ 1 presented the highest barrier against UVB (Figure 10a), UVA (Figure 10), and Total UV (Figure 10c). Besides that, St/MnO_2_ films exhibited the most progressive UV barrier (UVB, UVA, and UV total) with filler concentration increase: 0.25% (47.26 ± 1.32, 31.48 ± 1.38, 36.05 ± 1.36%), 0.5% (63.27 ± 0.69, 50.13 ± 0.93, 53.93 ± 0.86%), and 1% (81.88 ± 0.22, 74.49 ± 0.15, 76.62 ± 0.17%).

**Figure 10:**
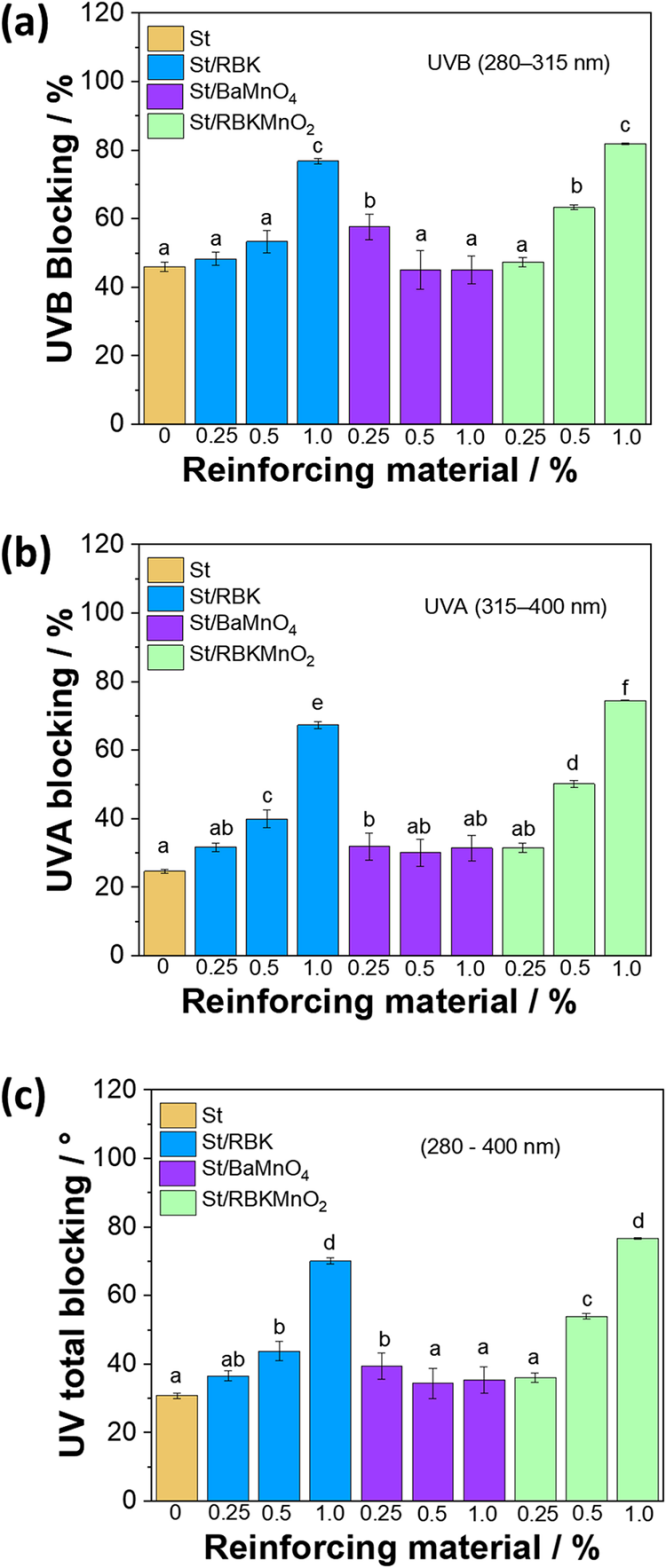
UV-blocking efficiency of starch films and composites. (a) UVB blocking (280–315 nm), (b) UVA blocking (315–400 nm), and (c) Total UV blocking (280–400 nm).

The results reflect the combined effects of absorption and scattering. MnO_2_, a dark transition metal oxide, contributes strong near-UV/visible absorption and interfacial scattering, explaining its leading performance at ≥0.5%. RBK (mineral residue) primarily increases scattering with some absorption. BaMnO_4_ behaves as a moderately dispersive, weakly absorbing filler in the near-UV. The optical response covaries with other properties: the most opaque films (RBK 1%, MnO_2_ 1%) also show the highest UV-blocking, indicating shared microstructural origins (absorbing domains, refractive index mismatch, and interface density).^58^

In order to place our findings in the context of the literature, Table 3 shows starch-based films reinforced with conventional nanofillers (e.g., silica, clays, and metal oxides) alongside the results obtained in this work with Mn residues. As shown, RBK- and BaMnO_4_-based films improved tensile strength and barrier performance comparable to those reported for traditional fillers.

MnO_2_-based films, in turn, maintained higher flexibility while exhibiting the strongest UV-shielding effect, which is particularly relevant for packaging applications requiring protection against light-induced degradation. BaMnO_4_-containing films consistently improved the tensile strength and barrier properties, reinforcing their potential as effective reinforcing agents. Remarkably, films reinforced with RBK, a raw residue obtained directly from mining activity without further chemical treatment, also exhibited clear enhancements in mechanical strength and water vapor resistance. This result emphasizes the feasibility of using minimally processed residues as functional fillers, adding value to waste materials. These findings demonstrate that Mn residues can deliver functional enhancements comparable to or superior to those achieved with conventional nanomaterials. Beyond performance, this strategy highlights the valorization of mining residues as functional additives, transforming environmental liabilities into high-value resources within a circular economy framework.

## 4 Conclusions

In this work, starch-based films reinforced with manganese residues (RBK, BaMnO_4_, and δ-MnO_2_) were successfully prepared and characterized. Incorporating these residues led to significant modifications in film performance compared to neat starch. Mechanical analysis showed that the tensile strength of neat starch films (3.5 ± 0.7 MPa) increased with the addition of RBK (up to 5.2 ± 0.6 MPa) and BaMnO_4_ (4.9 ± 0.5 MPa), while MnO_2_-containing films exhibited lower strength (2.8 ± 0.4 MPa). In terms of elongation at break, neat starch films reached 38.6 ± 4.2% but decreased to 24.3 ± 3.1% with RBK and 21.5 ± 2.7% with BaMnO_4_, whereas MnO_2_-based films maintained higher flexibility (33.7 ± 3.8%). RBK (2.5 ± 0.5 × 10^–10^ kg m^–1^ s^–1^ Pa^–1^) and BaMnO_4_ (2.8 ± 0.5 × 10^–10^ kg m^–1^ s^–1^ Pa) films exhibited reduced values of WVP compared to neat starch (4.9 ± 0.9 10^–10^ kg m^–1^ s^–1^ Pa^–1^), indicating better barrier performance. Residue-containing films enhanced UV shielding (UVB > UVA) and showed a concomitant increase in the opacity. Among them, MnO_2_ provided the strongest attenuation (≈82% UVB; ≈75% UVA at 1%), while RBK at 1% also reached a high-blocking tier (∼77% UVB; ∼67% UVA). Overall, these results confirm that the use of manganese residues can tailor the functional performance of starch films: RBK and BaMnO_4_ act as effective reinforcements for strength and barrier properties. At the same time, MnO_2_ maintains a higher flexibility and stronger UV protection. Beyond the functional improvements, this approach highlights the potential of valorizing mining residues within a circular economy strategy, coupling environmental sustainability with advanced materials design.
